# Supplementary material for: Digestate Improves Stinging Nettle (Urtica dioica) Growth and Fiber Production at a Chlor-Alkali Site
Source: Plants (Basel). 2024 Aug 30;13(17):2425. doi: 10.3390/plants13172425 (PMC11397052; doi:10.3390/plants13172425)
Supplement: Supplementary file 1 [file plants-13-02425-s001.zip › plants-3111121-supplementary.pdf]

Digestate Improves Stinging Nettle (*Urtica dioica*) Growth and Fiber Production at a Chlor-Alkali Site

Chloé Viotti [1], Coralie Bertheau [1], Françoise Martz [2], Loïc Yung [3], Vincent Placet [4], Andrea Ferrarini [5], Flavio Fornassier [6], Damien Blaudez [3], Markus Puschenreiter [7] and Michel Chalot [1,8,9,*]

[1] Université de Franche-Comté, CNRS, Chrono-Environnement, F-25200 Montbéliard, France; chloe.viotti@univ-fcomte.fr (C.V.); coralie.bertheau-rossel@univ-fcomte.fr (C.B.)
[2] Production System Unit, Natural Resources Institute (Luke), Ounasjoentie 6, 96200 Rovaniemi, Finland; francoise.martz@luke.fi
[3] Université de Lorraine, CNRS, LIEC, F-54000 Nancy, France; loic.yung@uha.fr (L.Y.); damien.blaudez@univ-lorraine.fr (D.B.)
[4] FEMTO-ST Institute, Department of Applied Mechanics, Université de Franche-Comté, F-25000 Besançon, France; vincent.placet@univ-fcomte.fr
[5] Department of Sustainable Crop Production, Università Cattolica del Sacro Cuore, Via Emilia Parmense 84, 29122 Piacenza, Italy; andrea.ferrarini@unicatt.it
[6] CREA—Centro Viticoltura ed Enologia, Via Trieste 23, 34170 Gorizia, Italy; flavio.fornasier@crea.gov.it
[7] Institute of Soil Research, University of Natural Resources and Life Sciences, 1180 Vienna, Austria; markus.puschenreiter@boku.ac.at
[8] Université de Lorraine, Faculté des Sciences et Technologies, F-54000 Nancy, France
[9] UMR 6249 Laboratoire Chrono-Environnement, Pôle Universitaire du Pays de Montbéliard,
4 Place Tharradin, F-25200 Montbéliard, France
* Correspondence: michel.chalot@univ-fcomte.fr; Tel.: +33-3-81-99-46-76

Supplemental material

Table S1. Mean chlorophyll content and photosystem II activity (PSII) (n=10±SE) of *Urtica dioica* leaves after 83 days of cultivation depending on the nature of amendment (C: compost; D: digestate), rate (+; ++; +++) applied and soil (St-Symphorien-sur-Saône or Tavazzano). *Different letters indicate significant differences between treatments and soils for each variable (Kruskal–Wallis test, P<0.05).*

Soil	Treatment	Chlorophyll content	PSII activity
	Control	6.6±0.3*ab*	0.75±8.79^{e-3}*b*
	C++	5.5±0.3*c*	0.78±6.54^{e-3}*a*
St-Symphorien-sur-Saône	C+++	5.9±0.9*c*	0.76±4.37^{e-3}*ab*
	D+	5.7±0.2*c*	0.78±7.18^{e-3}*a*
	D++	7.5±0.3*a*	0.77±7.03^{e-3}*ab*
	Control	6.4±0.3*b*	0.77±9.54^{e-3}*ab*
	C++	5.3±0.2*c*	0.76±1.31^{e-2}*ab*
Tavazzano	C+++	5.1±0.2*c*	0.76±8.01^{e-3}*ab*
	D+	5.5±0.2*c*	0.77±7.24^{e-3}*ab*
	D++	6.9±0.3*ab*	0.78±8.59^{e-3}*a*

Table S2. Fiber properties from *Urtica dioica* grown on St-Symphorien-sur-Saône soil depending on the amendment (C: compost; D: digestate) and rate (++; +++) applied (n=3±SE).

Treatment	Fiber max diameter (µm)	Fiber min diameter (µm)	Wall thickness (µm)
Control	47±6	36±6	8±2
C+++	53±3	40±3	9±1
D++	47±1	34±2	7±1

Table S3: Mean enzymatic activities (n=10±SE) expressed in nanomoles of 4-MUF.g^{-1} or AMC.g^{-1} soil in St-Symphorien-sur-Saône and Tavazzano rhizospheric soils after 83 days of cultivation depending on the nature of amendment (C: compost; D: digestate) and rate (+; ++; +++) applied. * *indicates a mean significantly different from the control for each soil*

	St-Symphorien-sur-Saône					Tavazzano				
**Enzyme**	**Control**	**C++**	**C+++**	**D+**	**D++**	**Control**	**C++**	**C+++**	**D+**	**D++**
aryS	5.5±0.6	7.6±0.3*	7.5±0.3*	5.8±0.7	10.3±1.1*	6.4±0.6	3.8±0.2*	5.5±0.4	3.6±0.4*	4.9±0.4*
alfaG	1.2±0.2	1.8±0.2	1.3±0.1	1.2±0.2	0.9±0.1	1.0±0.1	1.1±0.1	1.7±0.2	1.2±0.5	0.7±0.3^{e-1}
betaG	4.8±0.9	3.3±0.3	3.0±0.4	3.4±0.3	3.4±0.4	4.3±0.7	3.1±0.4	7.0±0.9	3.5±0.4	3.5±0.3
alfaGAL	3.3±0.6	2.4±0.5	3.1±0.6	3.6±0.7	3.3±0.6	3.2±0.6	3.1±0.6	4.1±1.1*	3.0±0.5	3.6±0.4
betaGAL	1.5±0.2	1.9±0.2	1.5±0.1	2.0±0.2	1.3±0.1	3.2±0.5	1.8±0.1*	3.1±0.4	4.0±1.9	2.4±0.1
cell	3.0±0.2	3.3±0.2	3.6±0.1	3.4±0.3	4.1±0.2*	3.7±0.3	2.7±0.3	3.9±0.2	3.6±1.2	3.1±0.2
xilo	0.4±0.2	1.1±0.1*	1.0±0.1*	1.2±0.1*	1.8±0.3*	1.6±0.1	1.5±0.1	2.4±0.2*	1.4±0.2	2.0±0.2
uroni	0.5±3.1^{e-2}	0.7±0.1*	0.8±3.7^{e-2}*	0.8±0.1*	1.2±0.1*	3.3±0.2	2.3±0.1*	3.7±0.2	2.1±0.2*	2.2±0.1*
chit	3.5±0.4	4.0±0.2	4.9±0.4	6.5±0.8*	10.6±1.3*	9.2±0.9	7.0±0.3*	10.6±0.7	8.0±1.2	9.4±0.7
leu	22.2±1.8	29.2±1.1	33.2±1.5*	34.3±3.1*	46.3±4.1*	46.8±3.0	36.0±1.7*	54.1±2.5	34.6±4.1*	41.9±2.5
acP	15.3±1.4	22.7±1.2*	28.6±2.2*	15.9±1.6	35.6±3.5*	29.3±3.5	20.9±1.0*	28.7±1.6	17.5±2.4*	20.4±1.2*
bisP	34.9±3.2	49.4±2.3*	47.2±1.4*	36.7±2.8	51.2±3.9*	33.2±3.1	28.5±1.2	42.2±3.0*	22.3±2.6*	26.5±1.5*
piroP	0.9±0.6	3.0±0.7	4.8±0.2*	4.0±0.9*	6.7±0.6*	5.5±0.8	4.5±0.3	5.1±0.9	3.9±0.6*	4.9±0.3
alkP	193.6±21.1	289.0±19.0*	296.3±17.5*	254.0±29.1	346.3±45.8*	269.4±25.2	273.4±13.4	387.9±32.3*	213.6±33.7	282.4±20.4
inositP	0.8±0.1	1.0±4.0^{e-2}*	1.1±4.2^{e-2}*	0.8±0.1	1.1±0.1*	1.1±0.1	0.9±0.1*	1.5±0.1*	1.1±0.3	1.0±0.1
butyr	58.3±6.4	158.1±22.3*	259.2±22.1*	148.7±23.1*	293.5±37.7*	110.1±12.1	267.2±45.8*	458.5±37.1*	105.2±14.3	260.3±59.0*
nona	53.3±6.0	227.6±31.1*	384.4±42.6*	153.0±28.6*	366.0±92.9*	132.4±16.0	413.8±101.5*	655.8±64.0*	112.1±14.6	357.8±67.4*

Table S4: Soil pH at T0 and at the end of the experiment (T83) for the two soils depending on the nature of amendment (C: compost; D: digestate) and the rate applied (+; ++; +++). *Different letters indicate significant differences between treatments (Kruskal–Wallis test, P<0.05).*

Treatment	Time (days)	St-Symphorien-sur-Saône	Tavazzano
Control	0	8.0±0.1*c*	6.4±0.2*g*
Control	83	8.1±0.1^{e-1}*bc*	6.9±0.4^{e-1}*fg*
C++	83	8.1±0.1*bc*	7.3±0.4^{e-1}*de*
C+++	83	8.1±0.2^{e-1}*bc*	7.5±0.5^{e-1}*d*
D+	83	8.2±0.2^{e-1}*a*	7.2±0.1*ef*
D++	83	8.1±0.3^{e-1}*b*	7.1±0.1*ef*

Table S5: Properties of the compost and digestate used.

Parameter	Compost	Solid digestate
pH	8.3	9.3
OM (‰)	250	172
Total N (‰)	9.8	4.2
$N\text{-}NH_4$ (‰)	0.06	0.50
$N\text{-}NO_3$ (‰)	0.09	0.01
C/N	15	11
P_2O_5 (‰)	3.4	4.0

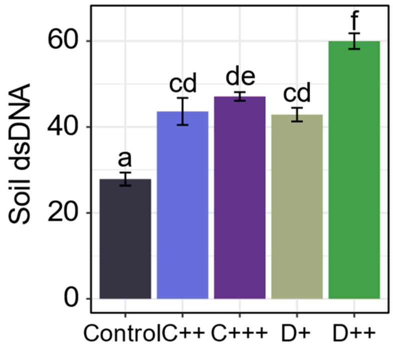

Figure S1: Mean soil dsDNA (n=10, ng.g^{-1}±SE) after 83 days of cultivation on the St-Symphorien-sur-Saône soil depending on the nature of amendment (C: compost; D: digestate) and rate (+; ++; +++) applied. *Different letters indicate significant differences between treatments (Kruskal–Wallis test, P<0.05).*

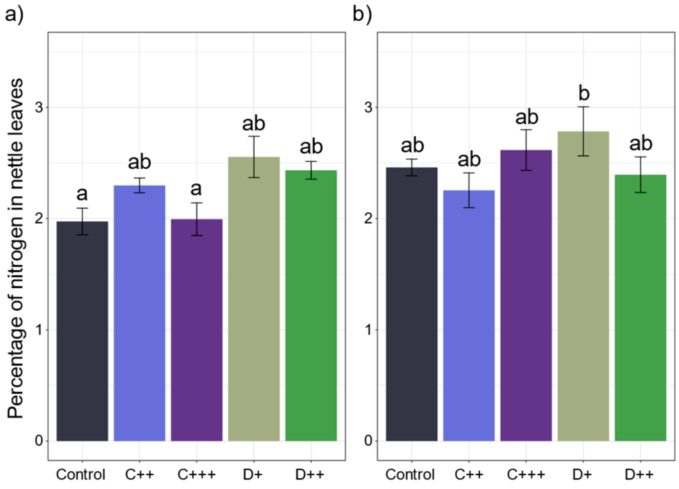

Figure S2: Mean percentage of nitrogen (n=5±SE) in *Urtica dioica* leaves after 83 days of cultivation on a) St-Symphorien-sur-Saône and b) Tavazzano soils depending on the nature of amendment (C: compost; D: digestate) and rate (+; ++; +++) applied. *Different letters indicate significant differences between treatments and soils (Tukey's test, P<0.05).*

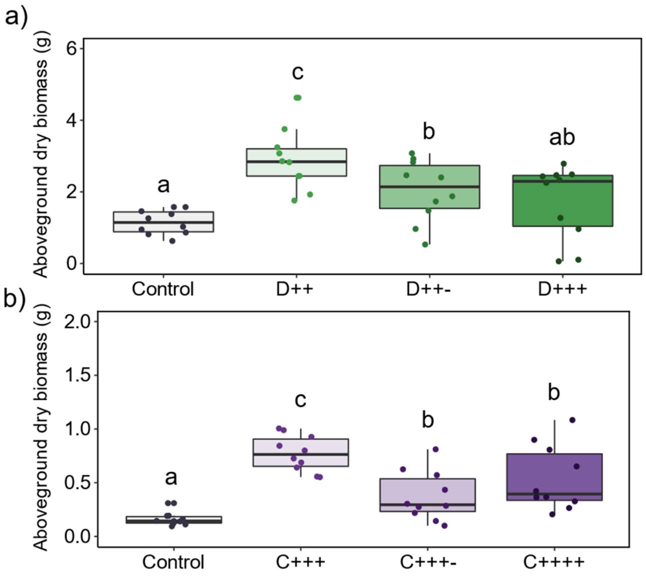

Figure S3. Aboveground dry biomass of *Urtica dioica* (n=10) in response to different rates (indicated by + and -) of a) solid digestate (D) and b) compost (C) in St-Symphorien-sur-Saône soil. *Different letters indicate significant differences between treatments (Kruskal–Wallis test, P<0.05).*
